# A Hybrid Mobile Phone Feasibility Study Focusing on Latino Mothers, Fathers, and Grandmothers to Prevent Obesity in Preschoolers

**DOI:** 10.1007/s10995-023-03700-w

**Published:** 2023-06-22

**Authors:** Guerrero AD, Glik DC, Jackson NJ, Whaley SE, Belin TR, W Slusser, Chung PJ

**Affiliations:** 1 UCLA Department of Pediatrics and Children’s Discovery and Innovation Institute, Los Angeles, CA USA; 2 Department of Community Health Sciences, UCLA Fielding School of Public Health, Los Angeles, CA USA; 3 UCLA Division of General Internal Medicine and Health Services Research, Los Angeles, CA USA; 4 Division of Research and Evaluation, Public Health Foundation Enterprises WIC, Irwindale, CA USA; 5 Department of Biostatistics, UCLA Fielding School of Public Health, Los Angeles, CA USA; 6 UCLA Semel Healthy Campus Initiative Center, Los Angeles, CA USA; 7 Department of Health Systems Science, Kaiser Permanente Bernard J. Tyson School of Medicine, Pasadena, CA USA

**Keywords:** Childhood obesity intervention, Latinos, Family, Mobile phones

## Abstract

**Objective:**

To pilot the feasibility of a mobile phone childhood obesity intervention for family caregivers of Latino preschool-aged children.

**Methods:**

An evidence-based early childhood obesity intervention was adapted to have cultural relevance and a shorter-length curriculum for mothers, fathers, and grandmothers of 2- to 5-year-old Latino children. Traditional in-person group sessions (four weeks) were combined with eight weeks of mobile phone content to support parenting skills and evidence-based and age-appropriate nutritional practices in either English or Spanish. A convenience sample of Latino families were recruited from WIC and Early Education Centers in East Los Angeles. Feasibility measures were collected. Child and caregiver height and weight were measured, and caregiver surveys of child dietary intake were collected at baseline, 1- and 6-month post-baseline. Changes in child’s dietary intake and BMI, as well as caregiver BMI, were examined using a mixed effects linear regression model with family random intercept and nested random slope for time period of measurement.

**Results:**

The program was delivered to 64 low-income Latino families (46 mothers, 34 fathers, 16 grandmothers, and 48 children). Children had a reduction in raw BMI, BMI percentile, and BMI z-scores at 6-months post-baseline compared to baseline measurements. The study also demonstrated stable BMI outcomes among all caregivers.

**Conclusion:**

The pilot study shows promise in preventing childhood obesity, and having a multi-generational impact on weight outcomes. Leveraging the high-use of mobile phones has the potential to shorten in-person interventions, and engage fathers and grandmothers who play an important role in shaping healthy weight practices in young children.

## Significance

*What is already known on this subject?* Obesity interventions focusing on caregivers show effectiveness in shaping healthy behaviors and weight outcomes for young children, including Latino children.

*What does this study ad?* Early childhood obesity interventions often focus on mothers, while mobile phone obesity interventions frequently focus on adolescents and school-aged children and are only provided in English. This study provides the feasibility and preliminary findings of a mobile phone intervention that focused on preschool children, and explicitly engaged multiple Latino family caregivers. The intervention considered language preferences (English or Spanish) allowing Latinos of varying levels of acculturation to participate.

## Introduction

One-quarter of American children are Latino and nearly half of all Latino children and youth are overweight or obese.(Skinner et al., 2016, 2018) Obesogenic behaviors that continue into adulthood, such as television viewing and consumption of sugar-sweetened beverages and unhealthy snacks, often begin in the first years of life.(Burdette et al., 2004; Sarker et al., 2015; Schwartz et al., 2010; Sicouri et al., 2018; Vanderloo et al., 2016) Parent interventions to prevent and manage childhood obesity, indeed have been successful in shaping these early practices in young children, including Latino children.(Ayala et al., 2010; Falbe et al., 2015; Ling et al., 2016; Slusser et al., 2012; Sung-Chan et al., 2013; Yun et al., 2015). Often these interventions focus on building authoritative parenting practices, changing behavior habits for children around diet, physical activity, media use, and sleep, altering the home environment to increase the availability of healthy foods, and by also supporting parents’ with their own behavioral habits. Many interventions, however, struggle to attain a modest sustained effect size on child BMI, with many studies demonstrating a small *(d = 0.08*) but significant effect size over the short-term (i.e. less than 6-months).(Yavuz et al., 2015).

Engaging multiple family members is one potential mechanism to improve the effectiveness of early childhood obesity interventions. More often than not, many early childhood obesity interventions focus on one parent, typically mothers. Excluding other caregivers in Latino families, however, overlooks an important opportunity to build concordance around healthy weight habits among families, as fathers, grandparents, and other caregivers can support or undermine maternal efforts to limit sugary-sweetened beverages and screen time, available food choices in the home, and restrictive and pressured child-feeding practices.(Davison et al., 2018; Enten & Golan, 2008; Guerrero et al., 2011, 2016; Patrick & Nicklas, 2005; Snethen et al., 2008) Successful inclusion of additional family members in Latino-focused interventions requires removal of common barriers of participation such as time, cost, transportation, and English-language proficiency.(Arai et al., 2015; Cason-Wilkerson et al., 2015; Ghai et al., 2014; Jensen et al., 2012; Reidy et al., 2012; Wong et al., 2013) The issue of time is particularly important to address across all US racial and ethnic groups, as many childhood obesity interventions are 3- to 4-months long, require weekly in-person sessions, and have demanding protocols.(Ayala et al., 2010; Ling et al., 2016; Slusser et al., 2012) Missing from the field of published childhood obesity interventions, therefore, are interventions that are pragmatic in regards to time and inclusive of family caregivers.

The use of mobile phones is one way to reduce these common barriers of participation. Mobile phones, particularly smart phones have been used to improve dietary, physical activity and sedentary behaviors. Most pediatric mobile phone interventions to date, however, have focused on English-speaking youth and adolescents.(Chen et al., 2017; Chen & Wilkosz, 2014; Fiks et al., 2017; Keating & McCurry, 2015; Militello et al., 2012; Poorman et al., 2015; Turner et al., 2015; Wickham & Carbone, 2015). Missing from the field are mobile phone interventions that focus on Latinos, Spanish language content, and preschool-aged children despite the fact that Latinos are high users of mobile phones (Arora et al., 2016) (Manganello et al., 2016) and experience a disproportionate burden of obesity, cardiovascular disease, and socio-cultural disparities. The focus of this feasibility study, therefore, was to incorporate and leverage the use smart phones to minimize common barriers of participation, reach Latino family caregivers, and engage them in childhood obesity interventions. This feasibility study, *Familias Unidas Ninos Sanos* (FUNS) program, adapted an evidence-based parent curriculum for low-income Latino parents of 2- to 5-year old children(Slusser et al., 2012) and delivered the content using traditional in-person sessions plus the use of smart phones (i.e. hybrid format) in both English and Spanish. The FUNS program was specifically developed for mothers, fathers, and grandmothers of 2- to 5-year-old Latino children. The content of the FUNS program incorporated the dietary, physical activity, and media-viewing behaviors recommended by the American Academy of Pediatrics guidelines, along with parenting skills to limit the use of food and sweets to manage children’s behavior.

The purpose of this study was to deliver the hybrid FUNS program to mothers and at least one additional caregiver (fathers or grandmothers) in either English or Spanish in order to assess: (1) feasibility, retention rates, uptake of mobile phone content, and participant satisfaction, and (2) the association between participation and children’s BMI and changing dietary behaviors. A secondary outcome of interest was whether the program could also decrease caregiver BMI.

## Methods

### Study Design

The FUNS intervention was evaluated using a pretest-posttest design to evaluate the impact of the program on dietary and physical behaviors, and parental and child BMI. Participants were enrolled between March and September 2017 and duration of participation was approximately 6 months. The FUNS program and pilot evaluation was approved the UCLA’s Institutional Review Board (IRB).

### Setting and Recruitment

A convenience sample of Latino families with children age 2- to 5-years was recruited from WIC and Early Childhood Education (ECE) Centers in the neighborhoods of East Los Angeles using introductory research flyers. Criteria to participate in the study included two caregivers of a young child who: (1) self-identified as an individual of Latino descent; (2) had a 2- to 5-year-old child/grandchild; (3) lived with or cared for the child/grandchild at least 20 h/week (where the relationship did not have to be biological); (4) spoke Spanish or English; and (5) had the ability and willingness to participate in the intervention and agreed to complete the baseline and post-baseline data collection protocols (this ability was determined by using a Subject Comprehension and Participation Assessment Tool provided by UCLA’s IRB). Adults who received services from WIC or ECE centers were provided with the research flyer. If there was interest in learning more about the study, a research team member would provide a brief overview and then screen the caregiver for the inclusion criteria. If the caregiver met the inclusion criteria, they were then asked to identify a second caregiver who could also potentially participate in the study based on the inclusion criteria. The second caregiver was then contacted to explain the study, confirm eligibility, answer questions, and review the informed consent process. The majority of the time, the first contact was made with the biological mother of the child, who then identified the child’s father or grandmother as the second caregiver who could potentially participate. The sampling frame was approximately 250 caregivers after all outreach strategies within WIC and local ECE centers were completed. Grandmothers not meeting the 20 h per week of caregiving responsibilities, was the most common reason a caregiver did not meet the inclusion criteria. (i.e. visits or cares for grandchild one day a week). About 80% of families from the sampling frame did meet eligibility criteria, but about half declined to participate and enroll into the study because the second caregiver (most often a father) could not meet the required time commitment of the study, and less often because they did not have an interest. Families were excluded if the child had medical conditions related to overweight status such as Prader-Willi Syndrome, taking weight loss medication, or was concurrently participating in a weight loss program. A total of.

### Familias Unidas Niños Sanos (FUNS) Program

**Fig. 1:**
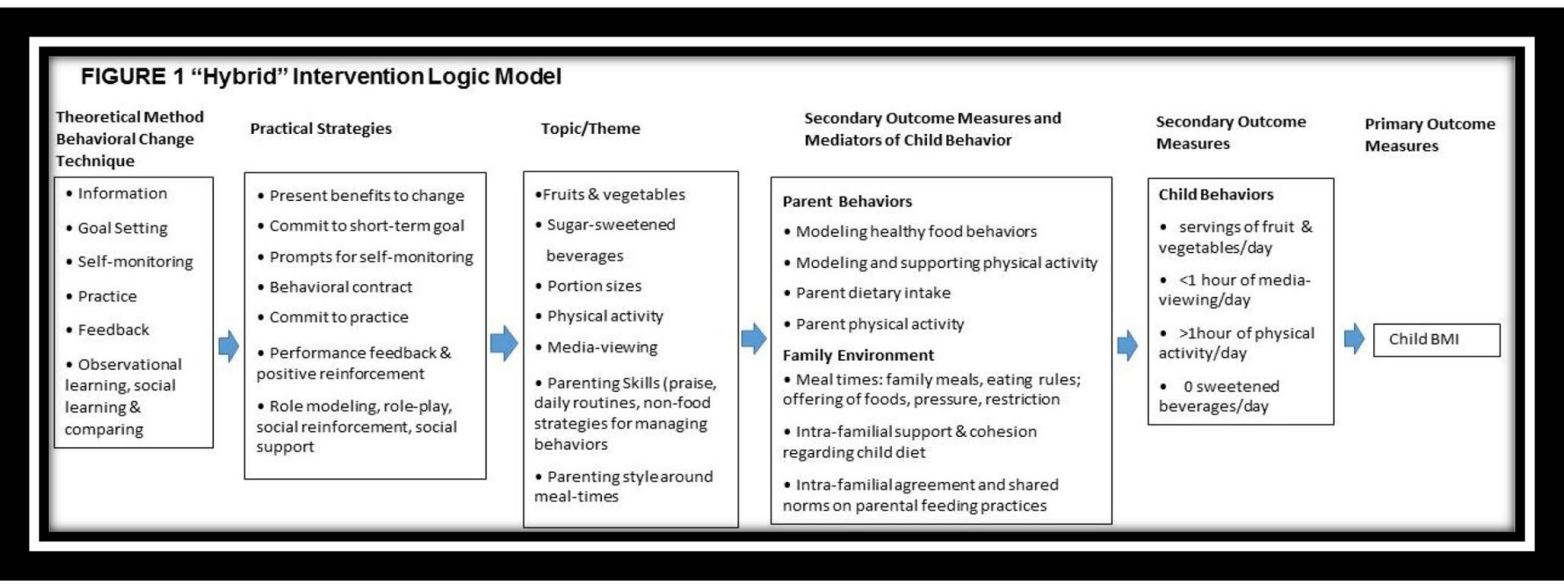
Logic model of intervention

The FUNS hybrid program is grounded in social-, cognitive-, and family-based theories and uses a group strategy to promote peer-to-peer learning, and observational and social support strategies to support behavioral changes. (Barlow & Expert, 2007; Davison & Birch, 2001; Golan & Weizman, 2001; Kitzman-Ulrich et al., 2010; Slusser et al., 2012; Sung-Chan et al., 2013) The FUNS hybrid program was developed based on an evidence-based parenting program to promote healthy weight (Slusser et al., 2012). It was adapted to emphasize the role of collectivism and familism among Latino families, and also informed by qualitative data collected from Latino mothers, fathers, and grandparents of two- to five-year old children/grandchildren to explore the challenges and barriers to support healthy dietary and physical activity behaviors. The knowledge and behavior goals for participants of the FUNS program, as reflected in the logic model in Fig. 1, focused on parenting skills and healthy-weight related practices consistent with the American Academy of Pediatrics (AAP) guidelines.(Barlow & Expert, 2007; Slusser et al., 2012) The FUNS program was delivered in either English or Spanish and included a 4-week program involving in-person plus mobile phone messages (hybrid approach).

Caregivers attended weekly 1-hour sessions facilitated by WIC staff. Participants who were mothers or grandmothers met together an attended 1-hour sessions weekly for 4-weeks. Those caregivers who were fathers, met in a group separate from mothers and grandmothers, and were only asked to complete the first two weeks of the in-person sessions that were facilitated by a male WIC staff member. (To increase father enrollment into the study, two weeks instead of all four weeks were required. Evening and Saturday sessions were also provided to encourage more father participation. Work schedules prevented many fathers from participating in all four sessions.). The two caregivers enrolled per child, (i.e. mothers, fathers, or grandmothers), however, received all 4-weeks of multimedia messages through their phones. Both the primary and secondary caregiver were sent the exact multimedia messages every week to introduce the topic of the week, prompt caregivers to practice a strategy, and provide practical tips on how to keep practicing. The multimedia messages included text, images, questions, and short video clips (two-to-four-minute video clips were developed in English and Spanish to support basic parenting skills and discourage the use of food or sweets to control a child’s behavior). After the 4-week hybrid program, both caregivers per child also received 8-weeks of “booster” messages via mobile phone only. The “booster” doses were created to reinforce the 4-week program content and support ongoing behavior changes. The “booster” messages included questions to assess information gained during the 4-week hybrid program, reminders to keep using the learned strategies and skills, and questions to assess the frequency of using learned strategies with their children.

**Fig. 2:**
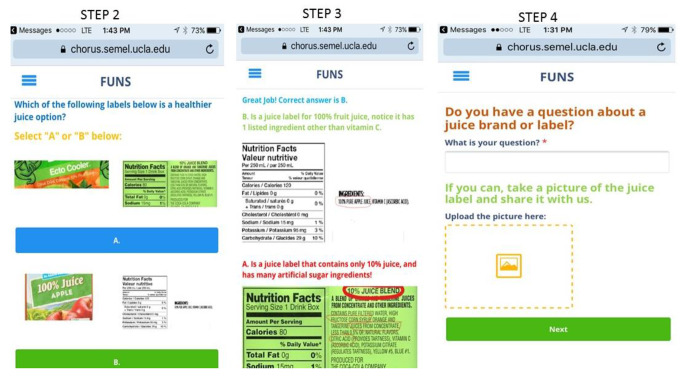
Example of the interactive multi-media content used in the web-based mobile phone application

*Chorus* was the mobile phone platform used to deliver the FUNS content.(A. C. Arevian, O’hora J, Jones F, Mango, J.D., Jones L, Williams P, Booker-Vuaghns J, Pulido E, Banner D, Wells K, 2018; A. C. Arevian, Springgate B, Jones F, Starks S, Chung B, Wennerstrom A, Jones L, Kataokoa S, Griffith K, Kascistis O, Haywood C, Kirlkand A, Myers D, Pasternak R, Simmaslsalm R, Tang L, Castillo E, Mahajan A, Stevens M, Wells, K, 2018) The *Chorus* platform provides a web-based application that can deliver simple text or multi-media messages without users having to download an application onto their phones.(Chorus Help Desk) Accessing the content without having to download a phone application was intentional and strategic in light of some evidence suggesting low-income and minority populations are high users of mobile phones, but may be less likely to use them to download applications. (Arora et al., 2016; Manganello et al., 2016) The web-based application was developed using existing *Chorus* platform templates to incorporate text, images, pictures, questions (open-ended and multiple-choice), short-video clips, or any combination of these. Users of the web-based application are able to access and interact with the multi-media content by clicking on a hyperlink embedded in a simple text message sent to their phone. The user interacts with the multi-media content by pressing “next” or “play” buttons, typing text into a field, clicking on an answer if there is a multiple-choice question, or uploading pictures by pressing “upload picture. Figure 2, provides a few examples.

### Training

The in-person sessions of the hybrid program were delivered by WIC health educators who are experienced and trusted health educators in their communities. A total of four health educators were trained to deliver the in-person sessions. A training manual was created for health educators that provided an overview of the program and included instructions on how to approach each session. The manual had the following typical layout for each session: (1) establish ground rules; (2) examples of open-ended questions and probes to facilitate discussion and peer-to-peer learning about the specific topic of the week, (3) caregiver handouts and activities for each week (informational pamphlets, role play cards, juice labels, etc.); and (4) examples of open-ended questions and probes to have caregivers commit to a weekly family goal related to the topic of the week. Collectively, this approach for every session supported the peer-to-peer learning, and observational and social support strategies to support the knowledge- and behavioral change-goals of the FUNS program.

### Measurements

Children’s and caregiver’s height and weight were measured by research assistants. Child dietary intake and the FUNS program’s family-centeredness were measured using surveys completed by the child’s primary caregiver (i.e. mothers) at baseline, 1-, and 6-month post baseline. Caregiver surveys were available in English and Spanish.

#### Child and Parent BMI

The NHANES Anthropometry Procedure was used by research assistants to measure child and parent height and weight and determine Body Mass Index (weight (kg)/height (m^2^). BMI percentile for age and gender was calculated using the CDC calculator and corresponding weight categorization (normal: ≥5% but < 85%; overweight: ≥85% but < 95%; obese: ≥95%).

#### Child Dietary Intake

The Children’s Dietary Questionnaire is a 28-item semi-quantitative questionnaire used to assess a child’s average consumption of four categories of foods or beverages. The questionnaire has been shown to have acceptable reliability and relative validity, and has been used among Latino preschool children in previous studies.(Magarey et al., 2009, 2011; Wiley et al., 2014) The questionnaire was available in English or Spanish for the child’s primary caregiver to complete. A few items were adapted if use of a non-colloquial US food was used in the question (i.e., “sweet biscuit” was adapted to pastries).

#### Family-Centeredness of mHealth Intervention Scale

The HRSA Child and Adolescent Health Measurement Initiative’s (CHAMI) family-centered subscale of the medical home was adapted for this study. The CHAMI family-centered subscale is used to assess whether children receive healthcare by a doctor who usually or always listens, spends enough time, is sensitive to family’s cultural values and customs, provides needed information, and makes the family feel like a partner in their child’s care. These items were adapted to evaluate the family-centeredness of the FUNS program such as, “During your participation in the FUNS program, how often did you feel recognized as the center of strength and support for your child?” and “During your participation in the FUNS program, how often did you feel supported to play a central role in your child’s care? A total of 9-items were used. See Appendix 1 for complete list of all 9 items.

### Statistical Analysis

Analyses of all data were completed using Stata 15.0 software (Stata Corp LP, College Station, Texas). Caregivers were designated “completers” if they attended ≥ 75% of classes, engaged with at least ≥ 1 mobile phone message per week, and completed baseline, 1- and 6-month assessments. Descriptive statistics (i.e., means and frequencies) of all outcome variables are presented. T-tests and chi-square tests were used to examine differences between completers and those who dropped out of the pilot on baseline characteristics. Among completers, baseline, 1-, and 6-month measures were analyzed. Changes in child or caregiver BMI over time were examined using a mixed effects linear regression model with family random intercept and nested random slope for time period of measurement. In instances where there was more than one child from a family participating in the study, the specification of the family random intercept resulted in averaging values across multiple children within a family in order to capture the average intervention effect at the family level.

## Results

### Participant Characteristics

The 6-month pilot study included 46 unique low-income Latino families. The families who participated included caregivers in the following combinations: mother-father, mother-father-grandmother, or mother-grandmother caregivers. The total sample, therefore, included 46 mothers, 34 fathers, 16 grandmothers, and 48 children (a few households had more than one child participating in the program). All caregivers enrolled in the study lived in East Los Angeles, and over 90% were of Mexican descent. Approximately 90% of the participants reported residing in low-income households and 75% of all caregivers received components of the intervention in Spanish. The average age of children in the study was 45 months, and approximately half were overweight or obese (Table 1).

**Table 1:** Caregiver and child characteristics at baseline

Characteristic	Completers	Dropouts	*P*
**Mothers** (caregiver-type), frequency	49	26	
Age (years), mean (± SD)	33.2 (± 5.4)	33.3 (± 6.14)	0.94
US born, %	40%	45%	0.68
<H.S. degree, %	34%	40%	0.64
Spanish-speaking home, %	73%	75%	0.89
**Fathers** (caregiver-type), frequency	40	13	
Age (years), mean (± SD)	36.7 (± 7.2)	39.1 (± 7.2)	0.26
US born, %	38%	15%	0.14
<H.S. degree, %	54%	20%	0.02*
Spanish-speaking home, %	75%	75%	0.99
**Grandmothers** (caregiver-type), frequency	18	4	
Age (years), mean (± SD)	57.1(± 7.9)	52.7 (± 8.5)	0.38
US born, %	11%	50%	0.07
<H.S. degree, %	77%	100%	0.35
Spanish-speaking home, %	75%	89%	0.46
**Children**, frequency	54	21	
Age (months), mean (± SD)	45.0 (± 12.8)	40.3 (± 11.3)	0.16
BMI Groups (age and sex matched), %			0.52
*Normal weight*	42%	53%	
*Overweight*	34%	37%	
*Obese*	25%	11%	
BMI percentile, mean (± SD)	75.6 (± 26.1)	70.7 (± 24.9)	0.48
BMI for age and sex Z-score, mean (± SD)	1.01 (± 1.01)	0.93 (± 1.47)	0.77

*denotes variables that differed significantly (< 0.05) between completers and dropouts

### Feasibility and Acceptability

Caregivers were considered “completers” if they attended ≥ 75% of the in-person sessions. The study’s retention rate at 1-month was 76% and 91% from 1-month to 6-months. There were no differences between completers and dropouts on socio-demographic factors or child’s weight status, with the exception of education among fathers (Table 1). Among the completers, 90% attended ≥ 75% of classes. In regards to completers interacting with the multi-media messages sent to their phones, during the 1st week 70% of all caregivers interacted with at least one message per week, followed by 68% during the 2nd week, 64% during the 3rd week and 67% during the 4th week. By caregiver type, 59% of mothers interacted with at least one message every week throughout the 4-week program, followed by fathers and then grandmothers at 51% and 45%, respectively. Approximately 90% of all caregivers who completed the 1-month post-baseline family-centeredness scale, endorsed usually or always to the family-centeredness of the FUNS program. **(**Table 2**)**

**Table 2:** Family centered experience with FUNS study

During your participation in the *Familias Unidas Ninos Sanos* program, how often did you feel the program usually or always…	All Caregivers N = 107	Moms n = 46	Dads n = 34	Grandmas n = 16
**Item**				
spent enough time focusing on you	89%	94%	85%	83%
provided needed information	93%	90%	90%	95%
recognized you as the principal caregiver	94%	94%	98%	89%
provided you clear and unbiased information and options to support healthy behaviors	95%	96%	93%	100%
supported you to play a central role in developing healthy behaviors and eating practices	96%	98%	95%	95%
gave you a shared responsibility in shaping your child’s/grandchild’s behaviors	94%	92%	95%	100%
recognized you as the expert in shaping your child’s/grandchild’s behaviors	86%	82%	88%	95%
was sensitive to your family’s values and customs	91%	92%	90%	89%
was sensitive to your family’s cultural strengths	89%	90%	90%	83%

Responses to each item assessed using a 1–4 Likert-scale: never, sometimes, usually, always

Column percentages are the proportion of responses indicating “usually” or “always”

### Impact

Improved dietary changes were noted among consumption of fruit and vegetables, sugar-sweetened beverages, and non-core foods (i.e., chips, fast food) at 6-months post-baseline compared to baseline measurements (Table 3). No significant changes over time were found in the intake of fat from dairy foods or milk. The child data at 6-months post-baseline revealed a reduction in child BMI compared to baseline measurements, whether examined by raw BMI, BMI percentile, or BMI z-scores (Table 4**).** Parental data at 6-months post intervention revealed stable weight outcomes among all mothers, fathers, and grandmothers (Table 4**).**

**Table 3:** Child dietary intake by parent report

Food Items	Possible Score range	Baseline mean score	6 Month mean score	Δ from Baseline to 6 Months mean (± SD)
**Fruits and Vegetables**	0–28	11.1	13.0	+ 2.17 (± 4.7)*
• # of fruits & vegetables last week				
• # of different fruits & vegetables last 24 h				
• frequency of fruits & vegetables last 24 h				
**Fat from Dairy**	0–15	4.1	3.5	− 0.66 (± 2.9)
• full fat milk last 24 h				
• full fat yogurt last 24 h				
• full fat cheese last 24 h				
**Sweetened Beverages**	0–5.9	1.7	1.2	− 0.50 (± 1.2)*
• frequency of fruit juice last 24 h				
• frequency of sodas in last week				
**Non-core Foods** Frequency last week divided by 7 of	0–10.3	2.0	1.5	− 0.54 (± 1.0)*
• sugary cereals				
• sugary yogurts				
• pan dulce/pastries				
• ice cream				
• chips				
• fast food				

*denotes significantly different (p < 0.05) change from baseline

**Table 4:** Child (n = 48) and Caregiver (n = 96) BMI Changes Over Time Using A Mixed Effects Linear Regression Model among Completers

	Baseline mean (± SE)	1 Month mean (± SE)	6 Months mean (± SE)
^1^Child Measures			
BMI raw score	17.4 (± 0.3)	17.2 (± 0.3)	17.1 (± 0.3)*
BMI percentile	74.8 (± 3.1)	72.7 (± 3.2)	70.2 (± 3.2)*
BMI z-score	0.99 (± 0.14)	0.91 (± 0.14)	0.82 (± 0.15)*
^**2**^ **Caregiver Measures**			
Mothers BMI (N = 46)	33.3 (± 1.0)	33.2 (± 1.0)	33.4 (± 1.0)
Fathers BMI (N = 34)	32.8 (± 1.0)	32.7 (± 1.0)	32.9 (± 1.0)
Grandmothers BMI (N = 16)	34.5 (± 1.6)	34.2 (± 1.6)	33.9 (± 1.6)

Mixed effects linear regression using family random intercept with nested person random intercept and random slope for time.

*denotes significantly different (p < 0.05) change from baseline

^1^Models adjusted for age at baseline, height, and sex

^2^Models adjusted for age at baseline

## Discussion

The pilot intervention demonstrates the feasibility of implementing a “hybrid” intervention using traditional in-person group sessions plus a mobile phone component among low-income bilingual Latino families with young children. The high usage of the mobile phone content in this study, 60–70% compared to 22–47% reported in other mobile phone interventions,(Hamine et al., 2015; Turner et al., 2015) demonstrates that Latino families have an interest and ability to use mobile phone content to support healthy weight behaviors for young children. The impact of the “hybrid-approach” intervention on child BMI also demonstrates the potential of integrating mobile phone applications to prevent childhood overweight and obesity using significantly shorter intervention periods, while simultaneously engaging fathers and grandmothers who are less often included, but play an important role in shaping dietary and physical habits in young children.

The study contributes to the feasibility of implementing family based-interventions, and offers a scalable methodology to deliver interventions that are typically challenging due to lengthy curricula and demanding protocols requiring weekly in-person sessions over a 2- to 6-month time period.(Ayala et al., 2010; Ling et al., 2016; Slusser et al., 2012) To our knowledge this is the first mobile phone application intervention focusing on Latino mothers, fathers, and grandmothers. Doing so addresses the role family members have in reducing the risk of childhood obesity through their direct or indirect influences on the home environment, as poor role modeling of dietary and physical activity patterns and poor intra-familial agreement around feeding practices are associated with childhood obesity risk.(Berge et al., 2016; Freeman et al., 2012; Halliday et al., 2014; Lubans et al., 2012; Semmler et al., 2009; Whitaker et al., 1997; Williams et al., 2018) In addition, including multiple family members provides an opportunity to leverage the culturally bounded value of *familismo* within Latino families to alter the prevalence of childhood obesity and cardiovascular risk. Addressing this cultural value with Latino populations has been shown to be important and effective in other behavioral health interventions.*(*Austin et al., 2013; Congello et al., 2020; Lescano et al., 2009; Ma & Malcolm, 2016*)*.

Feasibility and acceptability studies of mHealth interventions for Latino populations are important in order to identify those components that may work well to promote behavior change. Many MHealth pediatric obesity interventions (with and without a parent focus) use more than one modality to support behavior changes including internet, voice recognition, mobile phones (one-way or interactive texting), social media (such as Facebook or Twitter), telehealth, and email (Chen & Wilkosz, 2014) that may create limitations due to user proficiency with these modalities. In addition, many of the existing MHealth obesity interventions show effectiveness in changing healthy behaviors but primarily in adolescents and older adults. This study, therefore, contributes to a scant amount of literature on the feasibility of mobile phone interventions focusing on weight outcomes in preschool children delivered in both Spanish and English.

This study offers promising results for the use of a hybrid live plus mobile phone intervention to prevent obesity in Latin preschoolers. The pilot study supported healthier child dietary behaviors that were sustained for at least five months after the intervention. In addition, children had a reduction in child BMI, BMI percentile, and BMI z-scores at 6-months post-baseline compared to baseline measurements. Although the focus of the intervention was to support parents and family caregivers with knowledge and skills to promote healthy weight strategies for their young children, the study also demonstrated stable BMI outcomes among all caregivers. We did not measure caregiver dietary behavior changes, or other behaviors commonly associated with weight maintenance. It is plausible, however, that parents and family caregivers may have also changed their own dietary behaviors in order to role model healthier practices for their young children. Nevertheless, these caregiver BMI results, show promise for the intervention to have a multi-generational benefit, as most middle-aged adults typically have a 1lb to 2lb weight gain on a yearly basis.(Stenholm et al., 2015; Truong & Sturm, 2005).

There are several strengths and limitations to this study. The strengths of this study include the feasibility and acceptability of adapting and delivering components of an early childhood obesity intervention through a smart phone in either English or Spanish. Another strength of the study was the ability to engage Latino fathers and grandmothers into a mobile phone intervention. Some of the limitations of this study include lack of a comparison group to establish a strong relationship with the child and adult BMI findings. In addition, a weakness of the study was the use of a small convenience sample, therefore, deducing similar results in other groups may not be seen due to a selection bias. To determine a child’s average consumption of foods and beverages, the Children’s Dietary Questionnaire was used which was developed in Australia, but has also been used in the US among Latino children (Adams et al., 2022; Wiley et al., 2014). Lastly, engaging fathers to participate in the weekly 1-hour in-person sessions to the same degree as mothers was a challenge due to competing work schedules or a lack of interest. The study findings, nevertheless, have generated insights and preliminary data that will inform our future work using mobile phones on a larger scale.

There are few stand-alone mHealth interventions focusing very young children in the US, with none to date specifically including bilingual Latino parents and caregivers of preschool children. This pilot study has shown feasibility in merging the evidence-based components and strategies of both traditional in-person and mHealth parent-focused childhood obesity interventions in English and Spanish. In addition, the study leveraged the opportunities afforded by the high mobile phone ownership among Latinos in order to access effective early childhood obesity prevention strategies. Finally, the pilot study shows promise in preventing childhood obesity, and also having a multi-generational impact on weight outcomes. Future work will focus on testing the effectiveness of this intervention with a large randomized clinical trial.

## Data Availability

Data for this study are not publicly available.

## Code Availability

Available upon request.
